# Leeches as Sensor-bioindicators of River Contamination by PCBs

**DOI:** 10.3390/s90301807

**Published:** 2009-03-13

**Authors:** Stanislava Macova, Danka Harustiakova, Jitka Kolarova, Jana Machova, Vladimir Zlabek, Blanka Vykusova, Tomas Randak, Josef Velisek, Gorzyslaw Poleszczuk, Jana Hajslova, Jana Pulkrabova, Zdenka Svobodova

**Affiliations:** 1 University of Veterinary and Pharmaceutical Sciences Brno, Department of Veterinary Public Health and Toxicology, Palackeho 1-3, 612 42 Brno, Czech Republic; 2 Masaryk University, Faculty of Science, Research Centre for Environmental Chemistry and Ecotoxicology, Kamenice 126/3, 625 00 Brno, Czech Republic; 3 University of South Bohemia Ceske Budejovice, Research Institute of Fish Culture and Hydrobiology, Vodnany, Zatisi 728/II, 389 25 Vodnany, Czech Republic; 4 Szczecin University, Faculty of Natural Sciences, ul. Felczaka 3A, 71–412 Szczecin, Poland; 5 Institute of Chemical Technology, Department of Food Chemistry and Analysis, Technicka 5, 166 28 Prague, Czech Republic

**Keywords:** PCB indicator congeners, Skalice River, *Erpobdella*

## Abstract

The aim of the study was to evaluate the use of leeches of the genus *Erpobdella* as a means of assessing polychlorinated biphenyl contamination of watercourses. The River Skalice, heavily contaminated with PCBs, was selected as a model. The source of contamination was a road gravel processing factory in Rožmitál pod Třemšínem from which an estimated 1 metric ton of PCBs leaked in 1986. Levels of PCB were measured in leeches collected between 1992 to 2003 from 11 sites covering about 50 km of the river (the first sampling site upstream to the source of contamination and 10 sites downstream). The PCB indicator congeners IUPA no. 28, 52, 101, 118, 138, 153, and 180 were measured. Levels were highest at the four sampling sites nearest the source of pollution. The highest values of PCB congeners were found in 1992. PCB content decreased from 1992 to 2003 and with distance from the source. The study indicated that leeches of the genus *Erpobdella* are a suitable bioindicator of contamination in the surface layer of river sediments.

## 1. Introduction

Polychlorinated biphenyls (PCBs) were first synthesized in the late 19th century. Production spread rapidly with PCBs being used extensively in mechanical, chemical and electrical engineering among other industries. Because of their stability, resistance to degradation, and bioaccumulation, they persist over long periods of time, accumulating in plants and animals and entering the food chain [1–5]. In tissues of organisms, PCBs bioconcentrate at levels that exceed their concentrations in water, where levels may be below detectable levels. Organisms are therefore effective indicators of contamination levels because they reflect changes in the environment, and thus also in bioavailability of the substances and over long periods of time [2, 6–11].

Studies quantifying persistent organic pollutants (POPs) and metals levels in aquatic ecosystems typically focus on predators such as fish [12–21]. Fish are suitable for analyses because they are often the top predator in a food web, they accumulate a variety of contaminants, and their size is conducive to relatively rapid collection for analyses, and through human consumption they are linked with health issues [21,22]. However, the use of large fish as chemical bioindicators is of little use in shallow rivers where trophic levels are limited. In these situations, selecting a suitable indicator species is problematic.

**Figure 2.**
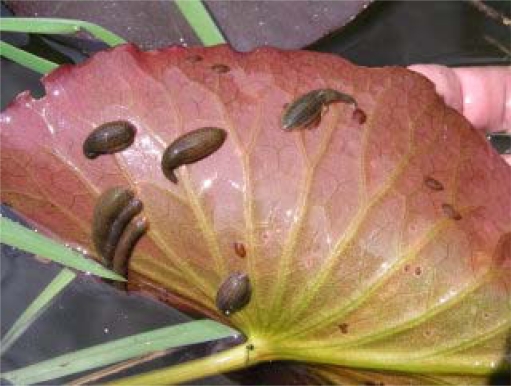
The main used bioindicator species - *Erpobdella octoculata.*

Certain features of leeches make them potentially useful as bioindicators of water pollution [23]. Leeches are among the most numerous animals in both standing and running freshwaters. There are several advantages of using the leech as an indicator organism. Leeches of the genus *Erpobdella* are abundant and easily accessible in small streams. Acquiring fish of similar species and age from different locations can be problematical. Leeches are residential organisms and better reflect conditions in the place from which they have been sampled than do fish, which with exceptions, migrate and may not have grown in the area where they are captured [24]. Compared to direct testing of sediments, leeches provide more objective data since they are taken from several tens of square meters at a given site. The most frequently occurring species at the site monitored was *Erpobdella octoculata* (Figure 2). *Erpobdella* spp. have a life span of 1–2 years, they live in flowing as well as stagnant water, and may be present in bodies of water ranging from oligosaprobic to polysaprobic in character. As predators, they feed on various species of aquatic invertebrates, mainly worms and midge larvae [25].

The Skalice River was heavily contaminated in 1986 with waste waters containing an estimated 1 metric ton of polychlorinated biphenyls that leaked from a road gravel processing factory in Rožmitál pod Třemšínem [26]. The aim of the study was to evaluate the use of the leeches of the genus *Erpobdella* as a means of assessing polychlorinated biphenyl contamination of watercourses. Monitoring for PCBs in the Skalice River began in 1992 and continued until 2003. Samples were collected from a single site upstream and at 10 locations downstream of the source of pollution covering a distance of about 50 km.

The following hypotheses were tested:

1. For each contaminated site and PCB congener: the content of PCB in fresh leech tissue is higher at the monitored site than at the control site.
2. For each site and PCB congener: the content of PCB in fresh leech tissue decreased at the site during the time period.
3. For each year and PCB congener: the content of PCB in fresh leech tissue decreased with distance from the source of pollution.

## 2. Material and methods

**Figure 3.**
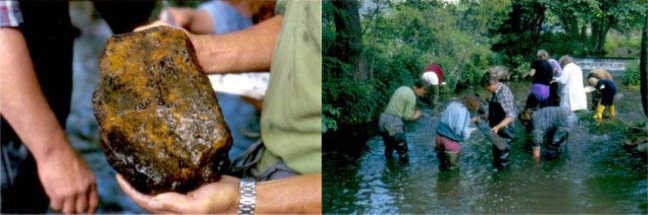
Collection of leeches in the Skalice River.

**Figure 4.**
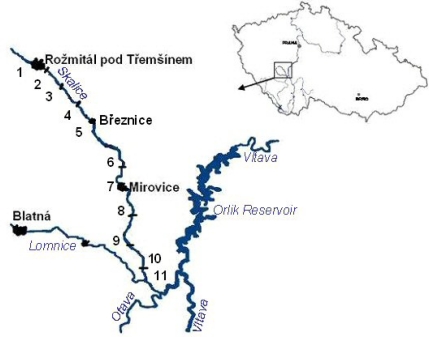
Locations of sites (Czech Republic).

Monitoring was carried out at 11 locations on the River Skalice between years 1992 and 2003 (Table 1, Figures 3,4). Sample site 1 was a control site located two km above the pollution source. Leeches of the genus *Erpobdella* (prevailing species *E. octoculata*) were collected every year except 2002. These collections were performed once a year in June. Due to the low abundance of leeches it was not possible to collect samples every year from each site. Leeches were dried slightly on filter paper, packed into microtone bags, immediately chilled, and frozen to −20˚C. One sample from each site weighed 50 g (corresponding to a composite sample of 600–700 leeches from one site together). This composite sample was analyzed.

A homogenous leech sample weighing 30 g was mixed with 100 g of anhydrous sodium sulphate to form a flowing powder and then extracted for 8 hours in a Soxhlet apparatus with 340 mL of a hexane-dichloromethane (1:1, *v/v*) solvent mixture. The crude extract was carefully evaporated and dissolved in 10 mL of a cyclohexane-ethyl acetate mixture (1:1, *v/v*) containing PCB 112 (this congener is not present either in commercial mixtures or environmental samples), employed as an internal standard.

A clean-up of crude extracts was carried out by an automatic gel permeation chromatographic system (GPC) employing S-X3 Bio Beads. As a mobile phase, cyclohexane-ethyl acetate (1:1, *v/v*) was used at a flow rate 0.6 mL/min and the fraction corresponding to the elution volume of 14–30 mL was collected. The eluate was evaporated by a rotary vacuum evaporator at 40°C and the residual solvent was carefully removed by a gentle stream of nitrogen. The residue was then dissolved in 1 mL of isooctane and treated with concentrated sulphuric acid to eliminate any co-extracted residues. An aliquot of the upper isooctane layer was transferred into a glass vial for following GC analysis.

An HP 5890 Ser. II, gas chromatograph (Agilent Technologies, USA) equipped with an electronic pressure control (EPC), a split/injector, two parallel 63Ni electron capture detectors (ECDs) and two parallel columns possessing a different selectivity (DB-5 and DB-17, both J&W Scientific, USA) were employed for all analyses of PCBs and OCPs.

GC conditions were as follows: splitless injection mode (splitless time: 2 min), injection volume: 1 μL, injector temperature: 280°C; DB-5 (5% phenyl-methylpolysiloxane) and DB-17 (50% phenyl-methylpolysiloxane) capillary columns (60 m × 0.25 mm × 0.25 μm); oven temperature program: 60°C (2 min), rate 30°C/min to 220°C, 0.5°C/min to 240°C and 2.5°C/min to 280°C (10 min). Temperature of both ECD detectors was 300°C. As a carrier gas, helium at constant flow 1.7 mL/min was used. Limits of quantification (LOQ - μg kg^−1^ lipids) are: PCB 28 – 0.5, PCB 52 – 0.6, PCB 101 – 1.0, PCB 118 – 0.6, PCB 138 – 1.0, PCB 153 – 0.6, PCB 180 – 7.

Values of PCB congeners were not normally distributed so nonparametric tests were used. The Wilcoxon matched pairs test was used to compare PCBs between control and downstream locations. Spearman rank correlation was calculated (Daniels test) to assess decrease in PCB levels over time as well as the decrease in PCB levels with distance from the source of pollution. Data analyses were performed using Statistica software (StatSoft Inc., 2007) [28].

## 3. Results

### 3.1. Comparison of PCB content at monitored sites with the control site Rožmitál

The concentrations of congeners PCB 28, PCB 52, PCB 101, PCB 153, PCB 180 and the total concentration of PCBs in leech tissue was significantly higher at the contaminated site Rožmitál, downstream of the source of pollution, than at the control site Rožmitál upstream. At Skuhrov (3), leech tissue showed significantly higher contents of PCB 28, PCB 52, PCB 101, PCB 118 and total PCBs than at the control site. At the next location downstream, Zadní Poříčí (4), the content of PCB 28, PCB 52 and total PCBs was significantly higher than at the control site. The highest values of PCBs were found at Březnice (site 5), which differed significantly from the control site in all PCB congeners and in total PCB content. At the next three sites (6: Myslín, 7: Mirovice, 8: Nerestce) neither total PCBs nor any individual congener differed significantly from the control site. The next location (Čimelice 9) differed significantly from the control site in PCB 28, PCB 52, PCB 153 and total PCBs. Content of PCB 28 and PCB 138 was significantly higher at site Ostrovec (10) than at the control site. The level of PCB 28 was higher at the final downstream site, the confluence with Lomnice River, than at the control site. However, there was no difference in total content of PCBs at the two most distant sites in comparison with the control site (Table 2).

### 3.2. Decrease of PCB content from 1992 to 2003

There was a significant decrease of PCB 180 at the control site during the period 1992–2003. The first two locations downstream of the source of pollution showed a non-significant decrease of most PCB congeners over time. Content of PCB 28, PCB 52, and total PCBs decreased significantly with time at site 4. Total PCB and all PCB congeners, with the exception of PCB 118 decreased significantly over the time period at location 5. Levels of PCB 28, PCB 52, PCB 101, PCB 180, and total PCB content decreased significantly over time at location 6. Concentrations of PCB congeners at site 7 decreased during the time period but not to a significant level. Levels of PCB 28, PCB 52, PCB 138, and total PCBs decreased significantly at site 8. Content of PCB 52, PCB 101 and PCB 180, but not total PCBs, decreased significantly over time at site 9. Total PCB, PCB 28, PCB 52, and PCB 180 decreased significantly over time at 10 (Table 3).

### 3.3. Decrease of PCB content with the distance from the source of pollution

Most of the PCB congeners decreased with the distance from the source of pollution, although not in every year and not always significantly.

**Figure 5.**
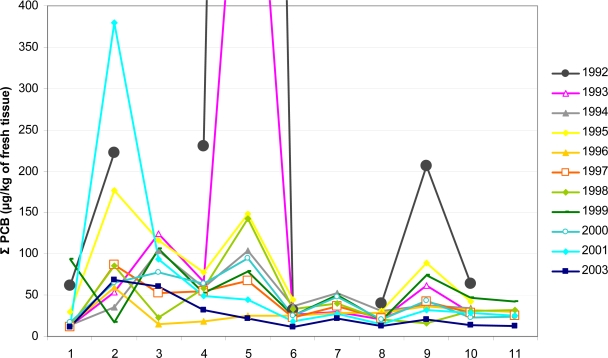
Σ PCB content in leeches from the River Skalice during 1992–2003. See table 1 for site codes. (Σ PCB content at site 5 in 1992 was 2763.0 μg.kg^−1^ in 1993 it was 767.0 μg.kg^−1^).

PCB 28 decreased significantly with the distance from the source of pollution only in 2003. PCB 52 decreased with the distance from the source of pollution nearly in every year, but the relationship was significant only in 1995, 1997 and 2003. PCB 101 decreased with the distance from the source of pollution nearly in every year, but the relationship was significant only in 1995, 1997, 2000, 2001, and 2003. PCB 118 decreased significantly with the distance from the source of pollution in 1997 and 2003. PCB 138 decreased with the distance from the source of pollution in every year, however the relationship was significant only in 1995, 1997, 2000, 2001, and 2003. Also PCB 153 decreased with distance from the source of pollution in every year, the decrease was statistically significant in 1992, 1995, 1997, 2000, 2001, and 2003. PCB 180 decreased with distance from the source of pollution in every year, however the relationship was significant only in 2001. Total PCB content decreased with distance of the source of pollution nearly in every year, but the relationship was significant only in 1995, 1997, 2000, 2001, and 2003 (Table 4, Figure 5).

## 4. Discussion

Benthic macroinvertebrates are moderately long-lived and are in constant contact with river sediments [8]. Contamination and toxicity of sediments will therefore affect those benthic organisms which are sensitive to them. Benthic macroinvertebrates are present, often abundantly, year-round, and since limited in mobility, reflect environmental conditions at the sampling point as well as changes over time and cumulative effects. Biological indicators can show problems otherwise missed or underestimated.

Some authors have investigated the use of suitable representatives of invertebrates as a sensors for the assessment of aquatic environment contamination with persistent organic substances [2,8]. A number have monitored the presence of leeches and concentrations of hazardous substances in their tissues to determine levels of water contamination, including organic contaminants and polychlorinated biphenyls [6,10–11,22–23,29–31]. Scrimgeour *et al*. [22] monitored contamination with selected persistent organic substances in the Beaver Hills Watershed (Alberta, Canada). PCB concentrations were determined in sediment samples and in leech tissue samples. In their case, environmental contamination levels were low and, in a majority of cases, below the detection limit.

Metcalfe *et al*. [10] assessed persistent organic compound concentrations in leech tissues and in water. Leeches were found to accumulate 16 of such compounds (two benzothiazoles, eight chlorophenols, lindane, and DDT and its derivatives), while only ten of the compounds were detected in water (benzothiazoles and chlorophenols). Accumulations of chlorinated phenolic substances in leech tissues exceeding levels in their aquatic environment have also been reported by Prahacs et al. [30]. Prahacs and Hall [11] found a high linear relationship between concentrations of chlorinated phenolic substances in water and bioconcentrations of those substances in leech tissue. This relationship is due to a slow elimination of chlorinated phenols, and it makes it possible to determine aquatic environment contamination levels from concentrations in leeches. Slow elimination of chlorophenols from, and a high degree of bioconcentration in, leech tissue has also been reported by Metcalf *et al*. [10] and Hall and Jacob [7]. The slow elimination increases biomonitoring sensitivity because larger amounts of hazardous substances are accumulated in leech tissue. Metcalfe *et al*. [29] found a high content of residues of chlorophenols in freshwater leeches when compared to fish, tadpoles, and other benthic invertebrates from an industrially polluted creek. Leeches may therefore serve as a suitable sensor-bioindicator of aquatic environment contamination with persistent organic substances.

### 4.1. Comparison of PCB content at monitored sites with control site Rožmitál – upstream to the source of pollution

The first hypothesis was a higher PCB concentration in tissue of leeches from contaminated sites compared with that from the control site upstream of the source of pollution. As expected, a comparison of concentrations of individual PCB congeners and the total of seven indicator PCB congeners in tissues from individual sites showed higher concentrations in the four sites nearest to the source of pollution. But the highest content of seven PCB congeners was surprisingly found in Březnice, i.e. the fifth site. It is assumed that this was caused by some other source of pollution believed to exist there.

### 4.2. Decrease of PCB content from 1992 to 2003

Variations in pollution levels over the period from 1992 to 2003 were also monitored. The highest levels of the congeners were found in the first year of monitoring, i.e. in 1992. In the period that followed, a majority of PCB congeners showed a decreasing trend in all monitored sites. A decrease in the total PCB content was observed in all sites with the exception of site 2, immediately downstream of the source of pollution. The decrease was not significant in all cases. It may be assumed that an important causal factor in the decrease was the superimposition of a new uncontaminated (or less contaminated) layer over the contaminated surface layer of sediment, or the washing away of contaminated sediment. Because leeches of the genus *Erpobdella* are bioindicators of pollution in the surface layer of sediment, the above processes will block the access of leeches to the underlying contaminated sediment layer and lead to a subsequent lower accumulation of PCB levels in their tissues.

### 4.3. Decrease of PCB content with the distance from the source of pollution

The third hypothesis was the decrease in PCB levels in leech tissues with distance from the source of pollution. A decrease, although not significant, was recorded for a majority of the PCB congeners monitored in each successive year of monitoring. Only a slight effect of contamination caused by the road gravel processing factory in Rožmitál pod Třemšínem is assumed in sites 6–11. These remote sites did not differ from the control site.

As representatives of macroinvertebrates, leeches occupy a certain position in the food chain of the aquatic environment. The relationship between the contamination of macroinvertebrates (i.e. leeches) and PCB levels in fish has been studied by a number of authors. Jackson *et al*. [17] compared PCB congener concentrations among fish (*Onchorhnychus kisutch* and *Oncorhynchus tshawytsha*) and macroinvertebrates in the Lake Michigan. While the representation of individual PCB congeners in macroinvertebrates and fish was comparable, there was a twenty to thirty-fold increase in total PCB content from macroinvertebrates to salmon. PCB concentrations in individual fish tissue, fish species, and in other aquatic organisms have been shown to be influenced to a large extent by fat contents, where PCB tend to accumulate [4,12,20–21]. In contrast, Zaranko *et al*. [5] and McCreanor *et al*. [31] found no significant relationship between lipid content and PCB concentration in leeches. Zaranko *et al*. [5], from 1989 to 1993, analyzed biota from Pottersburg Creek, Ontario, Canada for total PCBs and lipids. The relationship between PCBs and lipid suggested that organisms accumulate PCBs relative to their position in the food web [5]. Fish and leeches, which occupy the top of the food web, accumulated more PCBs than organisms occupying a lower trophic position. This indicates that biomagnification through trophic transfer (i.e., the uptake of a chemical through ingestion) is the primary mechanism governing contaminant levels in biota and not bioconcentration (i.e., the uptake of a chemical from water).

A different conclusion was drawn by DiPinto and Coull [32], who studied the dynamics of transfer of sediment-bound polychlorinated biphenyls from benthic copepods to juvenile fish. They found five time higher PCB accumulations in fish fed uncontaminated copepods and living in an environment with contaminated sediment than in fish in an uncontaminated environment fed contaminated prey. In analyzing PCB levels in zebra mussels (*Dreissena polymorpha*), Binelli and Provini [2] noted that bioconcentration played a role at the level of the first consumers (i.e. zebra mussel), while dietary uptake was important in organisms at a higher level of the food chain, including leeches. Comparing benefits of using either mussels (*Elliptio complanata*) or leeches (*Nephelopsis obscura*) as sensors of chlorphenol contamination, Metcalfe and Hayton [33] found leeches to be more suitable for the purpose.

The level of PCB contamination in the River Skalice was studied by Machala *et al*. [34] examining muscle tissue of two freshwater fish, roach (*Rutilus rutilus*) and chub (*Leuciscus cephalus*) caught in 1995 at nine sites along the River Skalice. The highest PCB concentrations were found downstream at Rožmitál and at Březnice. These results match those found in leech tissues.

The results obtained indicate that leeches of the genus *Erpobdella* are a suitable sensor-bioindicator of polychlorinated biphenyl contamination in rivers. PCB levels in their tissues reflect the current situation in contamination of the sediment surface layer and thus indicate levels of PCB that may be taken up by fish as the next link in the food chain.

## Figures and Tables

**Figure 1.**
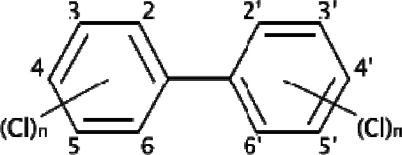
Polychlorinated biphenyls (PCBs).

**Table 1.** Sample sites on the River Skalice

**Site No.**	**Sample site location**	**Distance above confluence with R Lomnice (km)**
1	Upstream from Rožmitál pod Třemšínem - control site	45
2	Downstream from Rožmitál pod Třemšínem	43
3	Skuhrov	40
4	Zadní Poříčí	36
5	Březnice	32
6	Myslín	23
7	Mirovice	19
8	Nerestce	15
9	Čimelice	10
10	Ostrovec	2
11	confluence with Lomnice River	0

**Table 2.** Comparison of PCB content at 10 monitored sites with control site Rožmitál. Wilcoxon matched pairs test; p < 0.05 indicate significant difference from the control site (n = the number of years that leeches were sampled).

	**Sample site**	**n**	**PCB 28**	**PCB 52**	**PCB 101**	**PCB 118**	**PCB 138**	**PCB 153**	**PCB 180**	**Σ PCB**
**2**	**Rožmitál downstream**	11	**0.026**	**0.021**	**0.021**	0.076	0.066	**0.037**	**0.028**	**0.023**
**3**	**Skuhrov**	10	**0.005**	**0.008**	**0.012**	**0.014**	0.139	0.139	0.068	**0.005**
**4**	**Zadní Poříčí**	11	**0.006**	**0.006**	0.068	0.051	0.142	0.286	0.715	**0.010**
**5**	**Březnice**	11	**0.008**	**0.010**	**0.008**	**0.028**	**0.007**	**0.007**	**0.017**	**0.008**
**6**	**Myslín**	11	0.050	0.286	0.445	0.515	0.624	0.799	1.000	0.328
**7**	**Mirovice**	9	0.110	0.110	0.110	0.575	0.086	0.066		0.110
**8**	**Nerestce**	11	0.062	0.328	0.735	0.386	0.594	0.314	0.593	0.286
**9**	**Čimelice**	11	**0.003**	**0.005**	0.074	0.169	0.155	**0.038**	0.593	**0.008**
**10**	**Ostrovec**	11	**0.004**	0.155	0.484	0.333	**0.017**	0.080	0.593	0.050
**11**	**confluence with Lomnice**	7	**0.043**	0.237	0.463	0.866	0.080	0.116		0.237

**Table 3.** Statistical relationship of PCB content from 1992 to 2003 (n = the number of years when leeches were sampled, r_s_ Spearman rank correlation of time and PCB content, p - significance level).

**Site**	**n**		**PCB 28**	**PCB 52**	**PCB 101**	**PCB 118**	**PCB 138**	**PCB 153**	**PCB 180**	**Σ PCB**
**1**	11	r_s_	−0.144	−0.467	−0.337	−0.110	−0.038	0.070	−0.681	−0.321
		p	0.673	0.147	0.311	0.748	0.911	0.838	**0.021**	0.336

**2**	11	r_s_	−0.569	−0.583	−0.145	0.110	−0.073	0.228	0.075	0.073
		p	0.067	0.060	0.670	0.748	0.830	0.501	0.826	0.832

**3**	10	r_s_	−0.406	−0.442	−0.280	−0.293	−0.189	−0.043	−0.152	−0.370
		p	0.244	0.200	0.432	0.412	0.601	0.906	0.675	0.293

**4**	11	r_s_	−0.612	−0.724	−0.563	0.285	−0.501	−0.432	−0.296	−0.664
		p	**0.045**	**0.012**	0.071	0.395	0.116	0.184	0.378	**0.026**

**5**	11	r_s_	−0.645	−0.811	−0.618	0.000	−0.727	−0.627	−0.695	−0.755
		p	**0.032**	**0.002**	**0.043**	1.000	**0.011**	**0.039**	**0.018**	**0.007**

**6**	11	r_s_	−0.779	−0.839	−0.695	−0.065	−0.205	−0.156	−0.724	−0.743
		p	**0.005**	**0.001**	**0.018**	0.851	0.544	0.647	**0.012**	**0.009**

**7**	9	r_s_	−0.059	−0.395	−0.235	−0.194	−0.361	−0.142	−0.408	−0.367
		p	0.881	0.293	0.542	0.617	0.339	0.715	0.276	0.332

**8**	11	r_s_	−0.610	−0.849	−0.510	−0.288	−0.658	−0.167	−0.504	−0.781
		p	**0.046**	**0.001**	0.109	0.390	**0.028**	0.623	0.114	**0.005**

**9**	11	r_s_	−0.527	−0.636	−0.615	−0.174	−0.598	−0.114	−0.672	−0.582
		p	0.096	**0.035**	**0.044**	0.610	0.052	0.738	**0.024**	0.060

**10**	11	r_s_	−0.714	−0.680	−0.404	0.088	0.106	0.061	−0.732	−0.615
		p	**0.014**	**0.021**	0.218	0.798	0.757	0.858	**0.010**	**0.044**

**11**	7	r_s_	−0.630	−0.630	−0.206	−0.527	−0.236	−0.527	0.134	−0.577
		p	0.129	0.129	0.658	0.224	0.610	0.224	0.775	0.175

**Table 4.** Decrease in PCB content with distance from the source of pollution. (r_s_ Spearman rank correlation of distance from the source of pollution and PCB content, p - significance level). Only sites downstream of the source of pollution (numbers 2–11) were included in the analysis. (n =the number of sites where leeches were sampled)

**Year**	**n**		**PCB 28**	**PCB 52**	**PCB 101**	**PCB 118**	**PCB 138**	**PCB 153**	**PCB 180**	**Σ PCB**
**1992**	7	r_s_	−0.107	−0.667	−0.270		−0.739	−0.788	−0.579	−0.536
		p	0.819	0.102	0.558		0.058	**0.035**	0.173	0.215

**1993**	9	r_s_	−0.360	−0.613	−0.665	0.105	−0.502	−0.513	−0.137	−0.517
		p	0.342	0.079	0.051	0.788	0.168	0.158	0.725	0.154

**1994**	9	r_s_	−0.318	−0.613	−0.590	−0.392	−0.305	−0.244	−0.137	−0.485
		p	0.404	0.079	0.094	0.297	0.425	0.527	0.725	0.185

**1995**	8	r_s_	−0.452	−0.786	−0.838	−0.594	−0.778	−0.773	−0.394	−0.714
		p	0.260	**0.021**	**0.009**	0.120	**0.023**	**0.024**	0.334	**0.047**

**1996**	10	r_s_	0.152	0.188	0.024	0.030	−0.371	−0.042	−0.189	0.382
		p	0.676	0.603	0.947	0.934	0.291	0.907	0.600	0.276

**1997**	10	r_s_	−0.608	−0.707	−0.790	−0.796	−0.824	−0.813	−0.290	−0.697
		p	0.062	**0.022**	**0.006**	**0.006**	**0.003**	**0.004**	0.416	**0.025**

**1998**	10	r_s_	−0.347	−0.477	−0.447	−0.427	−0.448	−0.508	−0.174	−0.527
		p	0.327	0.164	0.196	0.219	0.194	0.134	0.631	0.117

**1999**	10	r_s_	0.164	0.141	−0.205	−0.092	−0.500	−0.272		−0.127
		p	0.650	0.698	0.570	0.800	0.142	0.448		0.726

**2000**	10	r_s_	−0.378	−0.579	−0.862	−0.189	−0.812	−0.784	−0.569	−0.782
		p	0.281	0.079	**0.001**	0.601	**0.004**	**0.007**	0.086	**0.008**

**2001**	10	r_s_	−0.494	−0.604	−0.661	−0.630	−0.925	−0.877	−0.792	−0.709
		p	0.147	0.065	**0.037**	0.051	**< 0.001**	**0.001**	**0.006**	**0.022**

**2003**	10	r_s_	−0.661	−0.636	−0.952	−0.709	−0.939	−0.927	−0.526	−0.758
		p	**0.038**	**0.048**	**< 0.001**	**0.022**	**< 0.001**	**< 0.001**	0.118	**0.011**
